# Towards the prediction of feed intake capacity of modern broilers on bulky feeds

**DOI:** 10.1016/j.psj.2021.101501

**Published:** 2021-09-24

**Authors:** James Taylor, Ilias Kyriazakis

**Affiliations:** ⁎ Agriculture, School of Natural and Environmental Sciences, Newcastle University, Newcastle on Tyne, NE1 7RU, UK; † Institute for Global Food Security, Queen's University, Belfast, BT7 1NN, United Kingdom

**Keywords:** adaptation, bulk, broiler, feed intake, water holding capacity

## Abstract

The use of alternative, often bulky ingredients is becoming widespread in poultry diets as the industry seeks to reduce its economic and environmental costs. Consequently, there is an increased need to accurately predict the performance of birds given such diets and identify their maximum capacity for bulk. We offered diets diluted with a range of bulky ingredients to male Ross 308 broilers to assess their capacity for bulk and identify a bulk characteristic responsible for limiting intake. Four hundred ninety-five day-old broilers allocated into 45 pens, were offered a common starter diet until day (d) 7, and 1 of 9 grower diets from d 8 to 29 (Period 1). Each of the grower diets was diluted with either 30 or 60% of oat hulls (**OH**), wheat bran (**WB**), or grass meal (**GM**), or a mixture of 2 bulky ingredients at an inclusion level of 30% each (OHWB, OHGM, WBGM). From d 29 to 43 (Period 2), all birds were offered the bulkiest diet (GM60). A number of bulk characteristics were measured on the diets. Feed intake was measured daily, and birds were dissected on d 29 and 43 for organ and carcass measurements. During d 8 to 14 diet water-holding capacity (**WHC**) was more consistent in predicting feed intake when scaled per unit of body weight than any other bulk characteristic. However, this was no longer the case during d 15 to 28. In Period 2, the response and adaptation to the bulkiest diet was determined by previous experience to bulk. Birds offered a bulkier diet during Period 1, were better able to adapt the size of their digestive organs and increase scaled feed intake, such that there were no differences between these birds and those offered the GM60; the converse was the case for birds on the least bulky diets. We conclude that WHC is able to predict maximum intake on bulky diets in unadapted birds. Adaptation to bulky diets can be very fast, so that their high bulk content no longer limits feed intake and performance.

## INTRODUCTION

Prediction of voluntary food intake is the basis of any simulation model that aims to account for bird performance under different management conditions (Kyriazakis and Emmans, 1999; Gous, 2007). The inclusion of alternative ingredients in poultry diets is becoming increasingly common to achieve sustainable food production and ensure global food security (Morgan and Choct, 2016; Tallentire et al., 2018; Tufarelli et al., 2018; Wyngaarden et al., 2020). As such there is an urgent need to understand and predict the ability of modern broiler genotypes to cope with such alternative ingredients, which are typically bulky in nature (Ravindran, 2013; Morgan and Choct, 2016; Scholey et al., 2020).

There have been concerns over the ability of modern broilers to modify their intake as the metabolizable energy content of the diet is reduced (Gous, 2013; Classen, 2017). However, there is now evidence to suggest that modern broiler genotypes have indeed retained the ability to regulate energy intake, as the energy content of the diet is reduced (Linares and Huang, 2010; Nascimento et al., 2020; Taylor et al., 2021). Clearly this ability is not infinite and holds only up to a maximum feed intake, which presumably relates to the maximum capacity of the gastrointestinal tract (**GIT**) for volume or bulk. Once this maximum GIT capacity is reached, energy intake will decline and bird performance will be reduced as the energy content of the diet is further reduced. The ability of broilers to cope with diets that are diluted with fibrous or bulky materials remains somewhat unclear. Two recent experiments provide contradictory evidence in quantifying a diet characteristic at which the maximum capacity for bulk intake and hence maximum feed or energy intake is reached (Nascimento et al., 2020; Taylor et al., 2021). Nascimento et al. (2020) offered diets diluted with a variety of bulky ingredients including cellulose fiber, rice husks, sawdust, vermiculite, and sand. They suggested that the water holding capacity (**WHC**, g water/g) of a diet could predict the maximum feed intake capacity of broilers, with the maximum scaled feed intake achieved on diets with approximately 2.6 g/g WHC. On the other hand, Taylor et al. (2021), suggested that the maximum scaled capacity for bulk could lie between 4.47 and 6.01 g/g WHC of a diet, but were unable to define the maximum scaled intake on diets that were progressive diluted with oat hulls or sugar beet pulp. In addition, Nascimento et al. (2020) did not find any adaptation to the bulky ingredients over time (for 45 d), whereas Taylor et al. (2021) suggested that adaptation on their bulky diets took approximately 15 d, although this depended on the nature of the bulky ingredient.

Any model that aims to accurately predict feed intake on bulky diets must be able to account for the rate of GIT adaptation to the bulky diets, as this will have direct implications on feed intake and by extension on performance (Whittemore et al., 2003). It is during this period of adaptation that feed intake and performance will be depressed to the greatest extent (Taylor et al., 2021). The rate of adaptation is seemingly dependent upon the physicochemical characteristics of the bulky ingredient (Taylor et al., 2021) and likely dependent upon the age at which birds are first introduced to the bulky diet (Jørgensen et al., 1996; Leeson et al., 1996; Sahraei and Shariatmadari, 2007). The ability to predict the extent to which intake and performance will be reduced, and for how long the period of adaptation will last after introduction to a bulky diet, will allow more accurate feed intake prediction for bulky foods to be developed, and may help to develop feeding strategies that will account or even minimize the effects of adaptation.

The objectives of this study were 3-fold: 1) to determine the capacity of a modern broiler genotype for bulky diets, which vary widely in their bulk characteristics, 2) to define a physical or chemical measure of bulkiness that accounts for the constraining effect of bulk on voluntary feed intake, 3) to determine the rate of adaptation to bulky diets. Furthermore, we hypothesized that feed intake on mixtures between bulky ingredients will result from the principle of additivity for dietary bulkiness, and that previous experience on bulky diets will improve the ability of birds to cope with a bulkier diet.

## MATERIALS AND METHODS

### Bird Management

All procedures were conducted under the UK Animals (Scientific Procedures) Act 1986 and approved by the AWERB of Newcastle University (no. 7332/2018). A total of 495 male Ross 308 chicks were obtained at day (d) 0 of age from a commercial hatchery and were housed in a thermostatically controlled building with 45 pens, each with an area of 0.85 m^2^. All birds were wing tagged upon arrival. Pens were equipped with feeders and drinkers, with wood shavings used as litter at a depth of 5 cm. The birds had free and continuous access to feed and water throughout the trial. The pen temperature was set to 34°C at arrival and was gradually reduced to 20°C by d 25 of age. The lighting schedule was 23 h Light (L):1h Darkness (D) for the first 7 d and was amended to 18L:6D for the course of the trial, whilst light intensity at pen level ranged from 80 to 100 lux.

Birds were individually weighed throughout the experiment; at arrival (d 0), prior to treatment allocation (d 8) and then twice per week until the end of the trial. After weighing the birds on d 8, the stocking density was reduced from 11 to 10 birds per pen. Chicks were then distributed between pens in a randomized block manner, so that there were no significant differences in the mean body weight (**BW**) between treatments. Pen feed intake was measured from d 0 to d8, and then daily until the conclusion of the experiment on d 43.

### Experimental Design and Diet Formulation

All birds were fed the same conventional starter diet from d 0 to 7of age, when they were then offered 1 of 9 experimental bulky diets from d 8 to 28 of age (Period 1; Table 1). At d 29 all birds were switched to the same experimental bulky diet (see below), until the conclusion of the experiment on d 43 (Period 2).

**Table 1:** Ingredient composition, calculated and analyzed chemical composition of the grower diets^a^ offered from d 8 to d 28 of age to broiler chickens.

Ingredients (%)	OH30	OH60	WB30	WB60	GM30	GM60^g^	OHWB	OHGM	WBGM
Ground maize	7.00	4.00	7.00	4.00	7.00	4.00	4.00	4.00	4.00
Ground wheat	35.2	17.4	35.6	22.4	32.8	12.7	19.9	15.0	17.6
Soybean meal (48% CP)	16.3	10.7	15.1	4.9	17.4	12.9	7.81	11.8	8.91
Full fat soya	4.20	2.40	4.20	2.40	4.20	2.40	2.40	2.40	2.40
Oat hulls (OH)	30.0	60.0	-	-	-	-	30.0	30.0	-
Wheat bran (WB)	-	-	30.0	60.0	-	-	30.0	-	30.0
Grass meal (GM)	-	-	-	-	30.0	60.0	-	30.0	30.0
Limestone	1.09	0.71	1.20	0.95	1.28	1.08	0.83	0.90	1.02
Monocalcium phosphate	0.90	0.57	1.43	1.60	1.48	1.72	1.09	1.15	1.66
L-Lysine HCL	0.31	0.32	0.30	0.30	0.48	0.65	0.31	0.49	0.48
DL-Methionine	0.30	0.24	0.29	0.23	0.38	0.41	0.24	0.33	0.32
L-Threonine	0.16	0.17	0.13	0.10	0.26	0.36	0.14	0.27	0.23
Valine	0.06	0.13	0.00	0.00	0.15	0.30	0.06	0.21	0.15
Soya oil	2.31	1.32	2.31	1.32	2.31	1.32	1.32	1.32	1.32
Salt	0.18	0.10	0.18	0.10	0.18	0.10	0.10	0.10	0.10
Sodium bicarbonate	0.11	0.06	0.11	0.06	0.11	0.06	0.06	0.06	0.06
Vitamin and mineral premix b	0.40	0.40	0.40	0.40	0.40	0.40	0.40	0.40	0.40
Titanium dioxide	0.50	0.50	0.50	0.50	0.50	0.50	0.50	0.50	0.50
Ronozyme c	0.01	0.01	0.01	0.01	0.01	0.01	0.01	0.01	0.01
Lignosulphonate	1.00	1.00	1.00	1.00	1.00	1.00	1.00	1.00	1.00
Total	*100*	*100*	*100*	*100*	*100*	*100*	*100*	*100*	*100*
Chemical composition (%) d									
Metabolizable energy (kcal kg^−1^) (calculated)	2,483	1,960	2,698	2,385	2,457	1,846	2,172	1,903	2,115
Crude protein (CP)	15.6	12.5	17.0	15.0	18.5	17.7	13.8	15.1	16.4
Lysine (calculated)	0.82	0.84	0.89	0.82	1.12	1.08	0.90	0.97	1.00
Crude fat (oil A) e	5.21	6.14	6.10	5.76	5.13	4.93	6.06	5.83	4.51
Total oil (oil B) f	5.64	6.84	6.13	7.02	5.67	5.42	6.54	6.40	5.09
Ash	4.60	5.20	7.90	8.10	9.50	11.50	7.00	7.80	7.10
Dry Matter	89.7	91.0	89.3	89.0	90.1	92.0	90.3	90.6	92.1
Calcium	0.72	0.63	0.79	0.71	0.88	0.86	0.61	0.69	0.71
Available phosphorus	0.50	0.42	0.49	0.49	0.62	0.59	0.45	0.51	0.56
DM digestibility	-	67.4	69.7	67.6	65.9	64.3	62.1	60.3	63.7
Crude fiber	6.70	12.2	6.40	9.30	12.3	16.7	10.7	14.6	13.4
Neutral detergent fiber	17.8	27.6	22.0	28.9	23.9	32.8	28.1	31.1	30.7
Acid detergent fiber	7.76	13.6	6.47	9.70	13.9	20.1	11.6	17.3	15.4
Acid detergent lignin	2.51	3.64	2.05	2.65	2.12	3.92	3.09	3.74	3.28
Diet density (g/ mL)	1.25	1.47	1.19	1.39	1.43	1.75	1.41	1.62	1.58
Water holding capacity (g/ g DM)	2.71	3.15	3.55	4.38	4.16	5.94	3.94	4.43	5.02

a The diets included either 30 or 60% of one of the bulky ingredients Oat Hulls (OH), Wheat Bran (WB) and Grass Meal (GM). Three additional diets were formulated by mixing two of the 60% bulky foods at a time: diets OHWB, OHGM and WBGM, respectively.

b Provided per kilogram of diet: Vitamin A (vitamin A acetate), 13.5kIU; Vitamin D (cholecalciferol), 5.0 kIU; Vitamin E (dl-α tocopherol acetate), 100 mg

c Blend of amylase and beta-glucanase.

d Analyzed composition unless otherwise stated.

e Ether extractable portion of fat.

f Total fat in the sample.

g All birds were allocated to the GM60 treatment from d 29 to 43 of age.

The starter diet followed breeder's recommendations and was identical to what was used by Taylor et al. (2021). A basal diet appropriate for the growing phase was formulated with 3,009 kcal ME/ kg and 22.0% digestible CP (Taylor et al., 2021) and was diluted with 60% of either Oat Hulls (**OH**), Wheat Bran (**WB**), or Grass Meal (**GM**) to produce three bulky diets: OH60, WB60, GM60, respectively. The chemical composition of the three bulky ingredients used is presented in Table S1. The ingredients were chosen for their substantial differences in the physical and chemical properties, consistent with experimental objectives. Each bulky diet had the same calculated digestible protein to AME ratio, and all other nutrient to AME ratios were the same as in the basal diet. Nutrient, including mineral ratios were maintained by increasing or decreasing synthetic amino acids, limestone and monocalcium phosphate inclusion, where appropriate. To increase palatability and binding of the pellets, a lignosulfonate pellet binder (Lignobond, Borregaard LignoTech AB, Sarpsborg, Norway) was included at 1%. All diets contained titanium dioxide (0.5%) as an indigestible marker.

Mixtures of the basal diet and the 3 bulkiest diets were created to produce a further 6 bulky diets, so all resulting diets had the same nutrient to AME ratios. The first mixture series was one-part basal and one-part bulky diets to produce a 30% dilution for each diluent: diets OH30, WB30, GM30. The second mixture produced the final 3 diets by mixing equal halves of 2 of the 60% level of inclusion diets: diets OHWB, OHGM, WBGM. Thus, the resulting mixtures contained 30% of each of the bulky ingredients. All 9 experimental diets (OH30, WB30, GM30, OH60, WB60, GM60, OHWB, OHGM, and WBGM) were offered in pellet form from d 8 to 28. The pellets were steam conditioned at 60°C before being passed through a 3-mm pellet mill die at a length of 9 mm. Each of the 9 bulky diets was replicated in 5 pens during Period 1 (d 8–28). Pens were allocated to dietary treatments in a randomized block manner.

After removal of a subset of birds for sampling on d 29, all remaining birds were switched to diet GM60, which was the bulkiest of the diets offered during Period 1. This allowed us to assess the rate of adaptation to a bulkier diet based on previous dietary bulk experience (objective 3). The birds remained on this diet until d 43, which was the conclusion of the experiment (Period 2).

### Sampling and Measurements

On d 29 and d 43 of age, 2 birds per pen with a BW close to the pen average were culled by intravenous lethal injection with sodium pentobarbital (Euthatal, Merial Harlow, United Kingdom). Birds had constant access to feed and water up to the point of euthanasia. Immediately following euthanasia all birds were weighed. The full GIT of each of the 2 birds per pen was removed and weighed in full. The lengths of the duodenum, jejunum and ileum were recorded, and a sample of digesta from each bird was obtained from the lower 2/3 of the ileum for digestibility analysis. The GIT was then separated into its individual components: crop, proventriculus, gizzard, duodenum, jejunum, ileum, ceca, and large intestine. Each of the components were weighed with its contents, before being carefully emptied of their digesta contents by gentle finger stripping to obtain empty weights. Empty carcass weight (**ECW**) was obtained by weighing the carcass with the feathers and head, but without the GIT. Empty body weight (**EBW**) was than calculated as BW (g) – gut fill (g).

#### Pre-cecal Digestibility

Analysis of digesta for titanium dioxide (**TiO**) concentration was performed according to the method of Short et al. (1996). Digesta samples were freeze-dried for 4 d before being stored at 4°C, pending analysis. A subsample of 0.1 g was placed in a microwave furnace at 600°C for 1 h until the sample was ashed and the ash weight was recorded. Following ashing, 10 mL of 7.4 M H_2_SO_4_ were added to the sample and placed in screw cap tubes and then microwave digested (CEM, MARS-5) for a further 1 h. Samples were then filtered through a Whatmann no.2 filter paper into 100 mL volumetric flasks, before 20 mL of hydrogen peroxide was added. The solution was then topped up to 100 mL with deionized water and shaken thoroughly. Then 3.5 mL of the solution were aliquoted to a curvette and ran through the spectrophotometer (Biochrom Libra S12) at 410 nm to measure the amount of light absorbance of the solution. Using a standard curve, the absorbance indicated the amount of TiO in the solution, which was then used to calculate the digestibility coefficient by the following equation:

DMdigestibility(%)=100−100×TiOinfeedDM/TiOinfaecesDM

#### Diet Analysis

All classical descriptors of bulk used traditionally in livestock research were measured. Some of these descriptors are highly correlated as they essentially measure the same bulk characteristics through different methods (Brachet et al., 2015). Samples of all diets were analyzed for crude fiber (Test method: Commission Regulation (EC) No.152/2009), acid detergent fiber (Test method: AOAC 973.18-1977), neutral detergent fiber (Test method: AOAC 2002.04-2005), and acid detergent lignin (Test method: AOAC 973.18-1977). In addition to the above measurements, all diets were analysed for metabolizable energy, crude protein, ash, dry matter, Calcium, Phosphorus, ether extract, and total oil. All analyses were performed at a UKAS accredited commercial laboratory to the internationally recognized standard for competence (Sciantec Analytical Services, Cawood, UK).

Water holding capacity analysis was performed in triplicate, using an adaptation of the Robertson and Eastwood (1981) method. A 1-g diet sample was soaked in 250 mL of H_2_O at room temperature for 24 h. Subsequently, the samples were filtered through a Whatman no. 1 filter paper and the wet weight of the samples recorded, before the samples were placed into an oven at 105°C overnight and the dry weight was recorded. Dietary WHC was then calculated as g of water/g of DM.

Diet density was determined in triplicate by the method described by Kyriazakis and Emmans (1995). First, 100 mL of distilled water at 37°C was placed in a 250 mL flask and a 50 g sample of pelleted feed was added. After mixing, a further 50 mL of water was added, and the contents allowed to equilibrate for 15 min before a final 50 mL of water was added. The sample was left to equilibrate for a further 15 min before the flask was filled to volume with water with a burette. The total amount of water contained in the flask was subtracted from 250 mL.

### Calculations and Statistics

Some birds on diet OH30 developed severe feather pecking early on in the experiment (by d 18) and this treatment was discontinued on welfare grounds; the observation was unique to this particular treatment, and therefore OH30 was not considered statistically due to incomplete data.

Average daily food intake (**ADFI**), average daily gain (**ADG**), and FCR were calculated over weekly intervals; data for Period 1 (wk 1–3) and Period 2 (wk 4–5) were analyzed separately. To account for a-priori differences in growth rate due to diet composition, ADFI and ADG data were scaled relative to the mean weekly BW (Whittemore et al., 2001). The scaled feed intake and daily gains were then transformed by the natural logarithm to ensure that the residuals were normally distributed and avoid statistical bias (Allison et al., 1995). A repeated-measures mixed model was implemented to analyze the transformed scaled feed intake for each week of Period 1, to assess whether broilers adapted to the different diets over time. A significant increase in the scaled maximum intake of each successive period would indicate that the birds were adapting to the given diet. The model included diet type (8 diets) and week as fixed factors, the two-way interaction between diet type and week, and pen as a random factor. For consistency, the transformed scaled ADG, and FCR from Period 1 was also analyzed in a repeated measures model which included diet type and week as fixed factors, the two-way interaction between diet type and week, and pen as a random factor.

Similarly, a repeated measures mixed model was implemented to analyze the transformed scaled feed intake, transformed daily gains and FCR for each week of Period 2; the model included diet type during Period 1 and week as fixed factors, the two-way interaction between previous diet type (8 diets) and week, and pen as a random factor. For both Period 1 and 2 analyses, covariance structures were chosen based on the lowest value for the Akaike and Bayesian information criteria.

Organ measurements were expressed relative to the EBW of the bird (g/ kg EBW) at the end of either Period 1 (d 29) or Period 2 (d 43) to account for the differences in growth performance (Oikeh et al., 2019). These data were analyzed with the general linear mixed (**GLM**) procedure with diet type as a fixed factor and pen as a random factor.

To evaluate which diet characteristics had the highest correlation with scaled feed intake, a principal component analysis (**PCA**) was used. For the PCA 11 variables (diet characteristics) were considered: crude protein, crude fat (Oil A), total oil (Oil B), dry matter, crude fiber, neutral detergent fiber, acid detergent fiber, acid detergent lignin, feed density, WHC, and indigestible matter (1-digestibility). A linear regression was utilized to assess the relationship between scaled feed intake against dietary WHC.

All statistical analysis was performed in R (Team, 2013), using the *factoextra* package and *prcomp* function to perform the PCA, and the *nlme* package and *lm* and *anova* functions for the repeated measures model, GLM procedures and linear regression (Pinheiro et al., 2014). For all statistical procedures, the normality of the residuals was assessed with qq-plots and the Shapiro-Wilk test; data did not need any further transformation. When significant differences were detected, treatment means were separated and compared by the Tukey's multiple comparison test. Significance was determined at *P* < 0.05.

## Results

### Diet Analysis

All measured characteristics of bulk increased when dilution levels increased from 30 to 60%. The crude fiber, neutral detergent fiber, acid detergent fiber, acid detergent lignin, feed density, and dietary WHC were greater in the GM60 diet compared to all other diets. The lowest crude fiber, acid detergent fiber, acid detergent lignin, and feed density were seen in the WB30 diet, whereas the lowest neutral detergent fiber and dietary WHC were seen in the OH30 diet. DM digestibility was greatest in the WB30 diet and lowest in the OHGM diet. There was additivity in all bulk characteristics when the diets were diluted with 2 bulky ingredients.

### Period 1

#### Feed Intake, ADG, and FCR

**Figure 1:**
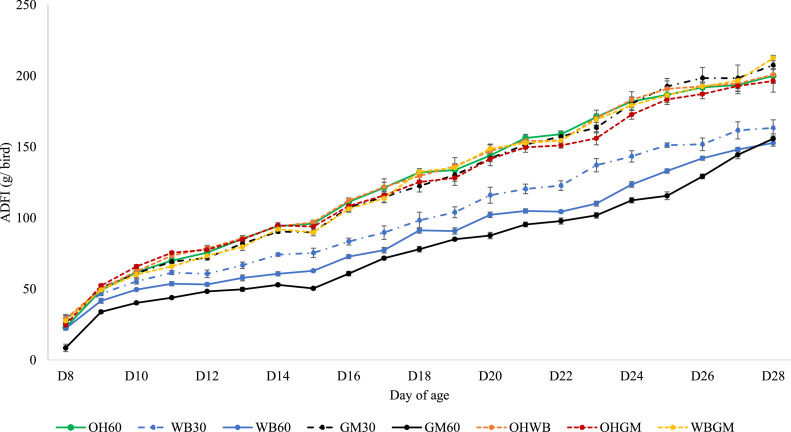
Average daily food intake (ADFI; g/ bird) of broiler chickens given access to foods containing Oat Hulls (OH at 60%), Wheat Bran (WB at either 30 or 60%), Grass Meal (GM at either 30 or 60%), or diets containing a mixture of two bulky ingredients at an inclusion level of 30% each (OHWB, OHGM, or WBGM), from d 8 to 28 of age. Each treatment was replicated in 5 pens containing 10 birds each.

The progression of daily feed intake from d 8 to 28 of age is shown in Figure 1 and the back transformed scaled feed intake (g/ kg/ day), scaled daily gains (g/ kg/ day) and FCR over wk 1 and wk 2 to 3 are presented in Table 2. There were no differences in the direction of the change in scaled feed intake between wk 2 and 3, and for this reason these weeks were considered together. There was a significant interaction between diet and week on the log transformed scaled feed intake (*P* < 0.05). The interaction was due to the differences in the direction of the change in the transformed scaled feed intake between treatments over time (wk 1 vs. wk 2–3).

**Table 2:** Average daily food intake and average daily gain expressed relative to the mean body weight of the time period (g/ kg/ day), and FCR calculated over d 8–14 (wk 1) and d 15–28 (wk 2–3) of broilers offered diets with different bulk contents*.

Diet type	ADFI/ BW (g/ kg/ day) ꝉ		ADG/BW (g/ kg/ day) ꝉ		FCR ⱡ		
	WK1	WK2–3	WK1	WK2–3	WK1		WK2–3
OH60	198^ab^ (185–214)	162cde (151–174)	101^b^ (94.6–108)	79.8^cd^ (74.4–85.6)	1.98		2.02
WB30	178^bc^ (165–191)	140^f^ (129–150)	105^b^ (98.5–112)	75.9^de^ (70.8–80.6)	1.85		2.06
WB60	164^cd^ (153–176)	164^cd^ (153–176)	85.6^c^ (79.8–91.8)	64.1^f^ (59.7–68.7)	1.87		2.03
GM30	181^bc^ (169–198)	140^f^ (130–150)	119^a^ (112–128)	79.8^cd^ (75.2–85.6)	1.73		2.28
GM60	144^ef^ (134–154)	154def (144–166)	80.6^cd^ (74.4–88.2)	68.7^ef^ (64.7–73.7)	1.81		2.09
OHWB	214^a^ (198–233)	166^cd^ (153–178)	100^b^ (93.7–107)	81.5^cd^ (75.9–87.4)	1.77		2.02
OHGM	212^a^ (196–230)	162^cd^ (151–176)	99.0^b^ (92.8–106)	78.3^cd^ (73.0–83.9)	1.92		2.26
WBGM	209^a^ (192–224)	169^cd^ (156–181)	105^b^ (97.5–111)	81.5^cd^ (75.9–86.5)	1.69		1.98
SEM					0.273		

	*Probabilities*						

Diet type	<0.001 <0.001 <0.001		<0.001 <0.001 <0.001			0.157 <0.001 0.428	
Week							
Diet type × Week							

⁎ Broiler chickens were given access to diets containing Oat Hulls (OH at 60%), Wheat Bran (WB at either 30 or 60%), Grass Meal (GM at either 30 or 60%), or diets containing a mixture of two bulky ingredients at an inclusion level of 30% each (OHWB, OHGM, or WBGM). Treatments were replicated in 5 pens containing 10 birds. OH30 treatment was discontinued on welfare grounds.

ꝉ Data were analyzed with a repeated measures mixed model, variables were analysed after transformation by natural logarithms and are presented here as back transformed means with confidence intervals.

ⱡ Data were analyzed with a repeated measures mixed model, variables presented as LS means and SEM.

a-f Means that do not share a common superscript are significantly different (*P* < 0.05) and represent the interaction between previous diet and week.

There was a significant interaction between diet and week on the log transformed scaled daily gains (*P* < 0.05). The scaled daily gains of the GM30 birds were significantly higher than all other treatments in wk 1, but were no longer significantly different from OH60, WB30, OHWB, OHGM, and WBGM birds in wk 2 to 3 (*P* > 0.05). There was no interaction observed on FCR (*P* > 0.05), but values in wk 1 were significantly lower (*P* < 0.001) than values in wk 2 to 3 across all treatments.

#### Relationship Between Scaled Feed Intake and Bulk Characteristics

**Figure 2:**
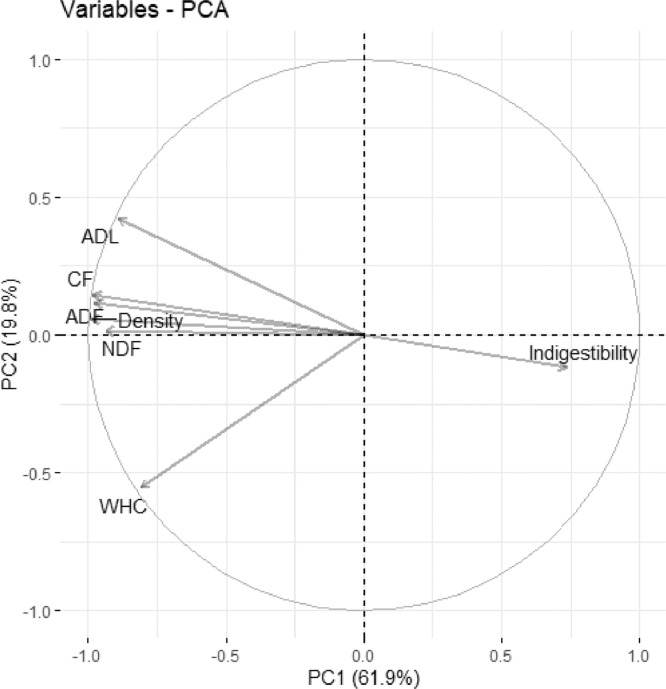
Variable correlation plots of scaled feed intake (g/ kg body weight) and bulk characteristics on the first two dimensions (PC1, PC2) of the principal component analysis (PCA) during wk 1 of the experiment (d 8–14). The bulk characteristics were: crude fiber (CF), acid detergent fiber (ADF), neutral detergent fiber (NDF), acid detergent lignin (ADL), water holding capacity (WHC), density and indigestibility. PC1 accounted for 61.9 % of the total variation and PC2 for a further 19.8% of the variation in the dataset.

**Figure 3:**
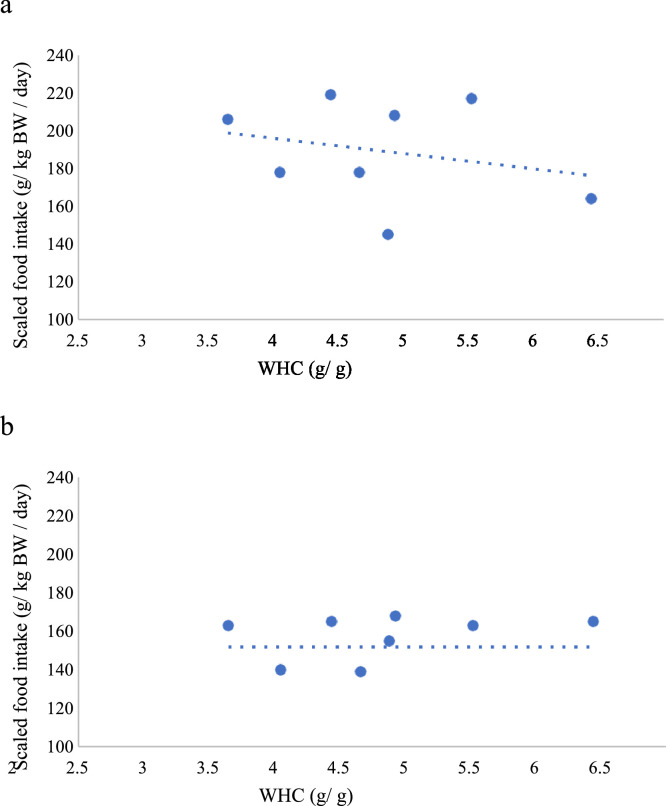
The linear relationships between scaled feed intake (SFI, g/ kg BW/ day) against the water-holding capacity (WHC, g/g) of the diets offered during Period 1: (a) the first week (d 8–14) and (b) wk 2–3 (d 15–28) of the period. For details of the diets offered in Period 1, see Table 1. The relationships were: (A) SFI = 224 (54.2) − 8.08 (12.32) WHC (*P* = 0.006), and (B) SFI = 137 (22.5) − 4.59 (5.12) WHC (*P* = 0.404). Each treatment was replicated in 5 pens each containing 10 birds.

Figure 2 shows the projections of the scaled feed intake and bulk characteristics on the first 2 dimensions of the PCA during wk 1. The analysis identified that PC1 accounted for 61.9% of the total variation and PC2 for a further 19.8% of the variation in the dataset. All fiber-related diet characteristics (CF, ADF, NDF, ADL), dietary WHC and diet density accounted for equal amounts of the variation within PC1, but the variable which was most highly correlated with scaled feed intake was dietary WHC (−0.483; *P* < 0.05). This relationship between scaled feed intake and dietary WHC is shown in Figure 3A. The correlation between dietary WHC and most fiber characteristics was high and positive (+ 0.711 to +0.739; *P* < 0.05), but moderate between dietary WHC and ADL (+ 0.477; *P* < 0.05).

Using data from wk 2 to 3, PC1 accounted for 60.2% of the total variation while PC2 accounted for a further 20.9% of the variation in the dataset (PCA projections not shown). Fiber characteristics accounted equally for the variation within PC1; however, the correlation between scaled feed intake and dietary WHC was weak and positive (+ 0.155; *P* > 0.05), shown in Figure 3B.

#### BW, EBW, ECW, and Gut Fill

The BW (g) of the dissected birds and their corresponding EBW (g), ECW (g), gut fill (g), and gut fill expressed relative to EBW (g/ kg) on d 29 are presented in Table 3; with the exception of gut fill, all other measurements were affected significantly by diet (*P* < 0.001). The lowest BW were seen on the WB60 and GM60 treatments, and the highest BW seen on the GM30 and OHWB treatments (*P* < 0.05); the BW of the latter 2 treatments were not significantly different between them (*P* > 0.05). BW on the remaining treatments lied between these extremes and was not always significantly different between them.

**Table 3:** Body weight (BW), empty body weight (EBW), empty carcass weight (ECW) and gut fill on d 29 of age of broiler chickens given access to diets of different bulk contents*. Gut fill was also expressed relative to EBW (g/ kg).

Diet type	Body weight (g)	Empty body weight (g)	Empty carcass weight (g)	Gut fill (g)	Gut fill (g/ kg EBW)
OH60	1,530^bc^	1,410^bc^	1,249^b^	121	77.3^c^
WB30	1,366^c^	1,245^c^	1,111^bc^	121	97.7^bc^
WB60	998^d^	881^d^	797^d^	115	134^b^
GM30	1,719^a^	1,613^a^	1,417^a^	106	66.8^c^
GM60	931^d^	786^d^	648^d^	144	184^a^
OHWB	1,560^ab^	1,436^b^	1,262^ab^	124	86.3^c^
OHGM	1,383^c^	1,253^c^	1,080^c^	127	103^bc^
WBGM	1,493^bc^	1,360^bc^	1,174^bc^	131	96.4^bc^
SEM	38.4	38.9	33.2	11.2	9.02

	*Probabilities*				

Diet type	<0.001	<0.001	<0.001	0.229	<0.001

⁎ Broiler chickens were given access to diets containing Oat Hulls (OH at 60%), Wheat Bran (WB at either 30 or 60%), Grass Meal (GM at either 30 or 60%), or diets containing a mixture of two bulky ingredients at an inclusion level of 30% each (OHWB, OHGM, or WBGM). Treatments were replicated in 5 pens containing 10 birds. OH30 treatment was discontinued on welfare grounds. Data were analyzed with the general linear mixed (GLM) procedure with diet as a fixed factor and results are presented as LS means with SEM.

a-d Means that do not share a common superscript are significantly different (*P* < 0.05) and represent the interaction between previous diet and week.

Consistent with the BW, birds given WB60 and GM60 had the lowest EBW and ECW (*P* < 0.05), and the birds on GM30 had the highest EBW and ECW (*P* < 0.05), although their ECW was not significantly different from OHWB birds (*P* > 0.0*5*). Birds on treatments OH60, WB30, OHGM, and WBGM had intermediate EBW and ECW, which were not significant between them (*P* > 0.05). Although there were no significant differences in gut fill between any of the treatments (*P* > 0.05), scaled gut fill relative to EBW was different between treatments: scaled gut fill of GM60 birds was significantly greater than any other treatment (*P* < 0.05) and scaled gut fill was greater in birds given WB60 than those offered OH60, GM30, and OHWB diets (*P* < 0.05). There were no further significant differences in scaled gut fill (*P* > 0.05).

#### Empty Organ Measurements Scaled Relative to Empty BW

Organ measurements scaled relative to EBW (g/ kg) on d 29 are presented in Table 4. Diet offered affected significantly relative GIT organ weight (*P* < 0.001). Birds on GM60 had significantly higher relative weights for any section of the GIT compared with birds on any other treatments (*P* < 0.05), with the exception of relative crop and ceca weights which were arithmetically, but not always significantly higher than all other treatments (*P* > 0.05). The treatment with the second highest relative organ weights was WB60, but its values were not always significantly different from all other treatments. The treatment with the lowest values of relative organ weights was GM30, whose values for all measurements were significantly lower than those of GM60 and significantly lower than those of WB60 for crop, gizzard, jejunum, and ileum relative weights only (*P* < 0.05).

**Table 4:** Organ (empty) weights on d 29 of age expressed relative to empty body weight (EBW, g/ kg). Broiler chickens given were access to diets of different bulk contents*.

Diet type	Crop empty (g/ kg EBW)	Proventriculus empty (g/ kg EBW)	Gizzard empty (g/ kg EBW)	Duodenum empty (g/ kg EBW)	Jejunum empty (g/ kg EBW)	Ileum empty (g/ kg EBW)	Ceca empty (g/ kg EBW)	Large intestine empty (g/ kg EBW)
OH60	6.98abc	5.98^b^	27.1^cd^	8.83^bc^	17.3^bc^	14.6^bc^	7.65^ab^	10.1^b^
WB30	6.43^bc^	8.09^b^	26.9^cd^	9.71^b^	19.9^bc^	15.3^bc^	7.03^b^	10.4^b^
WB60	9.11^ab^	8.21^b^	30.4^bc^	9.55^bc^	23.7^b^	17.8^b^	8.38^ab^	12.2^b^
GM30	4.61^c^	6.13^b^	18.8^d^	6.59^c^	13.6^c^	10.7^c^	5.87^b^	8.19^b^
GM60	9.25^a^	12.7^a^	42.4^a^	15.7^a^	33.5^a^	25.9^a^	10.4^a^	19.2^a^
OHWB	7.22abc	6.15^b^	27.3^cd^	8.53^bc^	18.9^bc^	14.1^bc^	6.36^b^	10.4^b^
OHGM	8.35^ab^	7.46^b^	28.1bcd	8.98^bc^	20.7^bc^	15.8^bc^	7.56^ab^	10.9^b^
WBGM	7.16abc	7.38^b^	26.6^cd^	9.63^b^	20.0^bc^	15.9^bc^	7.65^ab^	11.6^b^
SEM	0.585	0.559	2.33	0.636	1.65	1.36	0.724	1.046

	*Probabilities*							

Diet type	<0.001	<0.001	<0.001	<0.001	<0.001	<0.001	<0.001	<0.001

⁎ Broiler chickens were given access to diets containing Oat Hulls (OH at 60%), Wheat Bran (WB at either 30 or 60%), Grass Meal (GM at either 30 or 60%), or diets containing a mixture of two bulky ingredients at an inclusion level of 30% each (OHWB, OHGM, or WBGM). Treatments were replicated in 5 pens containing 10 birds. OH30 treatment was discontinued on welfare grounds. Data were analyzed with the general linear mixed (GLM) procedure with diet as a fixed factor and results are presented as LS means with SEM.

a-d Means that do not share a common superscript are significantly different (*P* < 0.05) and represent the interaction between previous diet and week.

### Period 2

#### Feed Intake, ADG, and FCR

The progression of daily feed intake from d 29 to 42 of age is shown in Figure 1 and back transformed scaled feed intake (g/ kg/ day), back transformed scaled daily gains (g/ kg/ day) and FCR over wk 4 and wk 5 are presented in Table 5. There was a significant interaction between previous diet and week during Period 2 on the log transformed scaled feed intake (*P* < 0.05). The interaction was due to the differences in the direction of the change in transformed scaled feed intake between treatments over time (wk 4 vs. wk 5). The transformed scaled feed intake of the birds previously offered GM60 was significantly different from all other previous treatments during wk 4 and 5 (*P* < 0.05), with the exception of the intake during wk 5 of the birds previously offered WB60 *(P* > 0.0*5*).

**Table 5:** Average daily food intake and average daily gain expressed relative to the mean body weight of the period (g/ kg/ day), and FCR calculated over d 29–35 (wk 4) and d 36–42 (wk 5). Broiler chickens were previously given access to diets of different bulk contents* before being offered a diet containing 60% grass meal (GM) from d 29 to 43 of age.

Previous diet type	ADFI/ BW (g/ kg/ day) ꝉ	ADG/BW (g/ kg/ day) ꝉ			FCR ⱡ		
	WK4	WK5	WK4	WK5	WK4		WK5
OH60	106^f^ (98.5–114)	128cde (118–140)	44.0 (37.3–51.9)	36.2 (31.2–42.1)	2.85		3.66
WB30	107^f^ (96.5–117)	126cde (114–137)	51.0 (42.5–62.2)	34.1 (28.8–40.4)	2.84		3.72
WB60	128cde (118–140)	159^ab^ (147–172)	40.0 (34.5–46.5)	30.3 (26.0–35.2)	2.91		3.86
GM30	105^f^ (97.5–112)	129cde (119–140)	56.0 (48.9–65.4)	36.6 (31.5–42.5)	2.37		3.36
GM60	174^a^ (159–189)	172^a^ (159–187)	37.0 (31.8–42.9)	30.6 (26.6–35.5)	2.86		3.98
OHWB	113^ef^ (104–123)	129^cd^ (119–141)	43.0 (37.0–49.9)	39.3 (34.1–45.6)	3.02		3.96
OHGM	125cde (114–136)	136^c^ (125–148)	40.0 (34.1–47.9)	35.5 (30.6–41.3)	2.89		3.65
WBGM	130^cd^ (120–140)	140^bc^ (128–151)	47.0 (40.4–54.6)	37.3 (32.1–42.9)	3.27		3.49
SEM					0.398		

	*Probabilities*						

Previous diet type	<0.001 <0.001 0.004		<0.001 0.001 0.062			0.180 <0.001 0.922	
Week							
Previous diet type × Week							

⁎ Broiler chickens were given access to a food containing 60% grass meal (GM) from d 29 to 43 of age. Broiler chickens were previously given access to foods containing Oat Hulls (OH at 60%), Wheat Bran (WB at either 30 or 60%), GM (at either 30 or 60%), or diets containing a mixture of two bulky ingredients at an inclusion level of 30% each (OHWB, OHGM, or WBGM) from d 8 to 28 of age. Treatments were replicated in 5 pens containing 8 birds. OH30 treatment was discontinued on welfare grounds.

ꝉ Data were analyzed with a repeated measures mixed model, variables were analysed after transformation by natural logarithms and are presented here as back transformed means with confidence intervals

ⱡ Data were analyzed with a repeated measures mixed model, variables presented as LS means and SEM.

a-f Means that do not share a common superscript are significantly different (*P* < 0.05) and represent the interaction between previous diet and week.

There were no further significant differences in transformed scaled ADFI during wk 5 (*P* > 0.05). There was no interaction between diet and week on the log transformed scaled daily gains (*P* > 0.05) or FCR (*P* > 0.05), and no significant effect of previous diet on FCR in either week (*P* > 0.05). The lowest log transformed scaled daily gains were seen in GM30 during wk 4 and in WB30 during wk 5 (*P* < 0.001).

#### BW, EBW, ECW and Gut Fill

The BW (g) of the dissected birds and their corresponding EBW (g), ECW (g), gut fill (g), and gut fill expressed relative to EBW (g/ kg) from d 43 are presented in Table 6; with the exception of gut fill, all other measurements were affected significantly by previous diet (*P* < 0.001). The BW of the birds previously on WB60 and GM60 were significantly lower than those previously on any other diets (*P* < 0.05) and the highest BW were seen on the birds previously fed GM30 and OHWB treatments, although these were not significantly different from the other birds (*P* > 0.05). Consistent with the BW, the birds previously given WB60 and GM60 had the lowest EBW and ECW (*P* < 0.05) and the birds previously on GM30 had the highest EBW and ECW (*P* < 0.05) although the latter were not significantly different from the other birds (*P* > 0.05). Although there were no significant differences in gut fill (*P* > 0.05), scaled gut fill relative to EBW was significantly different. The scaled gut fill was greatest in birds previously on GM60 (*P* < 0.05). However, there were no significant differences in scaled gut fill between the other birds (*P* > 0.05).

**Table 6:** Body weight (BW), empty body weight (EBW), empty carcass weight (ECW) and gut fill on d43 of age. Gut fill was also expressed relative to BW, EBW and ECW (g/ kg). Broiler chickens were previously given access to diets of different bulk contents* before being offered a diet containing 60% grass meal (GM) from d 29 to 43 of age.

Previous diet type	Body weight (g)	Empty body weight (g)	Empty carcass weight (g)	Gut fill (g)	Gut fill (g/ kg EBW)
OH60	2,556^a^	2,301^a^	2,062^a^	255	111^b^
WB30	2,543^a^	2,304^a^	2,056^a^	240	105^b^
WB60	1,988^b^	1,763^b^	1,566^b^	219	127^b^
GM30	2,647^a^	2,369^a^	2,132^a^	284	120^b^
GM60	2,008^b^	1,717^b^	1,493^b^	291	169^a^
OHWB	2,591^a^	2,337^a^	2,112^a^	253	108^b^
OHGM	2,380^a^	2,138^a^	1,919^a^	241	113^b^
WBGM	2,515^a^	2,239^a^	1,990^a^	276	124^b^
SEM	63.5	60.4	54.9	17.8	8.7

	*Probabilities*				

Previous diet type	<0.001	<0.001	<0.001	0.079	<0.001

⁎ Broiler chickens were previously given access to diets containing Oat Hulls (OH at 60%), Wheat Bran (WB at either 30 or 60%), GM (at either 30 or 60%), or diets containing a mixture of two bulky ingredients at an inclusion level of 30% each (OHWB, OHGM, or WBGM) from d 8 to 28 of age. Treatments were replicated in 5 pens containing 8 birds. OH30 treatment was discontinued on welfare grounds. Data were analyzed with the general linear mixed (GLM) procedure with diet as a fixed factor and results are presented as LS means with SEM.

a-b Means within a column that do not share a common superscript are significantly different (*P* < 0.05).

#### Empty Organ Measurements Scaled Relative to EBW

Organ measurements scaled relative to EBW (g/ kg) on d 43 are presented in Table 7. Previous diet affected all relative GIT organ weights with the exception of relative crop weight (*P* > 0.05). Birds that continued on the GM60 diet throughout the experiment had arithmetically, but not always significantly higher relative weights of section of the GIT (*P* > 0.05). The second highest relative organ weights were observed in the birds previously fed WB60, but the values were not always significantly different from the other birds. The lowest values of relative organ weights were observed in the birds previously fed WB30, with the exception of relative crop, duodenum, and ceca weights, which were lowest in the in the birds previously fed OHWB, GM30, and OHWB, respectively.

**Table 7:** Organ (empty) weights from d43 of age expressed relative to empty body weight (EBW, g/ kg). Broiler chickens given access to a food containing 60% grass meal (GM) from d29-43 of age. Broiler chickens were previously given access to diets of different bulk contents* before being offered a diet containing 60% grass meal (GM) from d 29 to 43 of age.

Previous diet type	Crop empty (g/ kg EBW)	Proventriculus empty (g/ kg EBW)	Gizzard empty (g/ kg EBW)	Duodenum empty (g/ kg EBW)	Jejunum empty (g/ kg EBW)	Ileum empty (g/ kg EBW)	Caeca empty (g/ kg EBW)	Large intestine empty (g/ kg EBW)
OH60	7.92	6.38^b^	23.2abc	7.98^b^	17.7^b^	14.3^ab^	5.70	10.4^ab^
WB30	6.78	6.56^ab^	19.7^c^	8.12^b^	19.0^ab^	14.5^ab^	5.77	8.67^b^
WB60	7.81	7.49^ab^	26.5^ab^	9.93^ab^	23.8^ab^	16.8^ab^	6.81	12.9^a^
GM30	7.62	6.73^ab^	20.6^c^	7.86^b^	19.7^ab^	13.7^b^	4.98	9.73^ab^
GM60	8.21	8.52^a^	27.2^a^	10.8^a^	24.2^a^	17.5^a^	6.85	13.3^a^
OHWB	6.71	6.58^ab^	21.5^bc^	8.11^b^	20.1^ab^	13.9^ab^	4.95	10.6^ab^
OHGM	7.70	7.35^ab^	23.3abc	8.20^b^	19.3^ab^	14.5^ab^	5.62	11.7^ab^
WBGM	7.18	6.90^ab^	21.4^c^	8.36^b^	19.2^ab^	14.8^ab^	5.29	12.1^ab^
SEM	0.510	0.448	1.08	0.42	1.28	0.905	0.457	0.819

	*Probabilities*							

Previous diet type	0.362	0.023	<0.001	<0.001	0.008	0.012	0.053	0.001

⁎ Broiler chickens were previously given access to diets containing Oat Hulls (OH at 60%), Wheat Bran (WB at either 30 or 60%), GM (at either 30 or 60%), or diets containing a mixture of two bulky ingredients at an inclusion level of 30% each (OHWB, OHGM, or WBGM) from d 8 to 28 of age. Treatments were replicated in 5 pens containing 8 birds. OH30 treatment was discontinued on welfare grounds. Data were analysed with the general linear mixed (GLM) procedure with diet as a fixed factor and results are presented as LS means with SEM.

a-b Means within a column that do not share a common superscript are significantly different (*P* < 0.05).

## DISCUSSION

We used 3 bulky ingredients (oat hulls, wheat bran, and grass meal) to investigate the capacity of a modern broiler strain for bulk, with the overarching objective of reaching a prediction of maximum capacity of the birds for bulky diets. This is of particular relevance in informing models and predictions of broiler feed intake and performance (Nascimento et al., 2020), especially now that there is an increased interest in the use of alternative, potentially bulky ingredients in broiler diets (Morgan and Choct, 2016; Scholey et al., 2020). Quantifying the capacity for bulk will define the level of inclusion of such ingredients and the energy density of a diet that will not penalise bird performance. The three bulky ingredients and their levels of inclusion covered a wide range of physiochemical properties, and as far as we are aware, they represent the highest level of inclusion (maximum 60% of the diet) of bulky ingredients used for broilers in the literature. We do not know which of the experimental diets limited feed intake and consequently the performance of the birds through its bulk, because the experimental design did not include a basal, non-limiting diet against which the intake and performance of the birds could be compared to. However, the performance measured, either as ADG or EBW, at the end of Period 1, was lower on all diets compared to birds on GM30. During the same period FCR was higher on all diets compared to GM30, with the exception of the WBGM mixture. Therefore, it can be safely assumed, that all diets, perhaps with the exception of GM30, limited intake and performance.

As expected, several of the physicochemical properties of the diets were highly correlated, as they essentially measured the same properties of the ‘fiber’ components of the diets (Brachet et al., 2015). In some cases, the correlations between the measurements between the ‘fiber’ component of the diet and physicochemical properties were broken up, as was the case between the ADL and dietary WHC. Consistently with the results of Nascimento et al. (2020) for broilers, the WHC of the diets was the physical characteristic of the diet most highly correlated with scaled feed intake. This correlation was moderate and is depicted on Figure 3A. The correlation did not improve when diet GM30 was excluded from the analysis. We have assumed, therefore, that the WHC of the diet is the physical characteristic of the diet that best represents its bulkiness. This is consistent with the suggestion made by Kyriazakis and Emmans (1995) and Tsaras et al. (1998) about the property of bulky diets best able to predict the maximum feed intake of pigs.

The WHC of a diet denotes the capacity of its fibrous component to trap water in its matrix, swell and form gels with high water contents (Eastwood, 1973; Ndou et al., 2013). This physical distension, or bulk increase, is carried throughout the digestive tract resulting in an increase in gut fill, delayed emptying of the GIT and consequently a reduction in voluntary feed intake (González-Alvarado et al., 2010; Jiménez-Moreno et al., 2013). In this respect, our results are consistent with the experiment of Nascimento et al. (2020) who suggested that the high retention of water by bulky ingredients can act as a limiting factor in various parts of the GIT. In terms of the range of the dietary WHC covered by our experiment (2.71 g/g–5.94 g/g for the OH30 and GM60, respectively), this is similar to the range covered by Taylor et al. (2021) for the same age and bird strain. The linear relationship between these 2 variables during wk 1 was:

$$\begin{array}{c} \text{Scaled}\;\;\text{feed}\;\;\text{intake}\;\;\left(g/\;\text{kg}/\;\text{day}\right)=768\;\;\left(s.e.\;\;23.7\right)\times \left(1/\text{WHC}\right)\\ \text{Residual}\;\;\text{Standard}\;\;\text{Deviation}\;\;34.3 \end{array}$$

Given the negative correlation between scaled feed intake and dietary WHC, it is suggested that the relationship between the two variables maybe of the form proposed by Tsaras et al. (1998) for pigs, which implies that the WHC of a diet can allow for accurate predictions of voluntary feed intake on bulky diets only when the diet has a constraining effect.

However, the above relationship between dietary WHC and SFI did not hold during wk 2 and 3 of Period 1 of the experiment, as there was essentially no relationship between the 2 variables (Figure 3B). It is possible that this was due to the adaptation of the birds on the diets offered, which may no longer have been limiting feed intake, at least for a number of the diets. This implies that birds were able to adapt at least to some of the diets relatively quickly and within a space of less than a week. This is logical when one considers the age at which the birds were introduced to the bulky diets and the greater plasticity of the GIT of young birds (Sklan, 2001). These outcomes are consistent with those of a previous experiment (Taylor et al., 2021), which suggest that in the same bird strain (Ross 308) birds were able to adapt to bulky diets based on oat hulls and sugar beet pulp, relatively rapidly, even after a week feeding on bulky diets that limited their intake and performance. In both the Taylor et al. (2021) and this experiment, the rate of adaptation to the bulky diet depended on the ‘bulkiness’ of the diet: in the former experiment birds offered a diet diluted with a high level of sugar beet pulp and high WHC, were unable to completely adapt to the diet by the end of the experiment.

On the other hand, Nascimento et al. (2020) gave Cobb 500 broilers access to diets which were progressively diluted by a variety of bulky ingredients, with a widely varying dietary WHC (ranging from 2.38 to 8.38 g/g). They suggested that their birds did not adapt to the high levels of inclusion of the bulky ingredients over a 45-d period, with the exception of the birds offered diets diluted with sand, which was one of the ingredients with the lowest WHC used. We do not have an explanation to offer for the discrepancy between the experiment of Nascimento et al. (2020) and the 2 Taylor experiments, other than the experiments were conducted on different bird strains and in the former case some of the birds were fed individually, whereas in the latter they were group-housed. However, the contrasting results between the experiments have significant implications: in the case of the Nascimento et al. (2020) they suggest that gut capacity of modern broiler genotypes may be only marginally extended when the nutrient density of a given diet is diluted, something also previously suggested by Tallentire et al. (2018), whereas the Taylor experiments suggested that the opposite is the case.

We tested whether there was an interaction between treatment and week to investigate the extent of adaptation to the diets. An interaction showing a significant increase in the scaled feed intake from wk 1 to wk 2–3 would indicate that the birds were indeed adapting to their respective diets, which was the case here. There was no difference in the scaled feed intake from wk 1 to wk 2–3 in the WB60 birds; meanwhile there was an increase in scaled feed intake in the GM60 birds, whereas there was a reduction in scaled feed intake for the remaining treatments. This indicates that the birds on these 2 treatments (WB60 and GM60) were undergoing a prolonged adaptation to the diets in comparison with the remaining treatments, which was expected as these were considered the 2 bulkiest diets. The adaptation period, in terms of scaled feed intake, was reflected in the relative GIT development of the birds. The highest dilution with grass meal (GM60) led to the highest relative GIT organ weights compared to all other treatments at the end of Period 1 (d 29); this was the case for every GIT component considered and it is consistent with the observation that this was the most limiting diet in relation to food intake and performance. The GIT development of the WB60 and GM60 birds accommodated their respective increases in scaled feed intake from wk 1 to wk 2–3, as the birds consumed feed to their evolving maximum bulk capacity. Consistent with our previous experiment (Taylor et al., 2021), the weight of the colon and the ceca was also significantly increased in the bulky treatments, confirming the frequently overlooked role of the large intestine in the accommodation of bulky diets (Amerah et al., 2009; Abdollahi et al., 2019). The relative weights of the GIT were higher in the birds given WBGM and OHGM rather than the OHWB, showing that there was an additive effect of bulkiness on the development of the GIT. In our previous experiment (Taylor et al., 2021), the relative GIT organ weights responded linearly to progressive diet dilution with either oat hulls or sugar beet pulp; this could not be tested in this experiment.

Following the change to the bulkiest diet (GM60) during Period 2, the relative weights of the crop and ceca of birds that were previously offered one of the other 7 diets in Period 1 were no longer different to the those birds that were offered the GM60 diet throughout both Period 1 and 2. It has been established that the physical use of the crop in chickens is reduced in modern broilers, since there is continuous access to feed in poultry systems (Classen et al., 2016). Furthermore, it has been suggested that the addition of structural components to the diet stimulates the crop and increases its development (Kierończyk et al., 2016). It is therefore possible that the birds in our experiment began to utilise their crop to a greater extent in Period 2 than Period 1, as they had to cope with a bulkier diet (GM60) than their original diets in Period 1. By the end of Period 2 (d 43 of age), the relative GIT organ weights were no longer different between previous feeding on GM60 and WB60. Similarly, the scaled feed intake of these 2 treatments was no longer significantly different. This suggests that the bulkiness of a previous diet can define how quickly the GIT adapts to a switch to a bulkier diet, as was the case here. Whittemore et al. (2003) suggested that the initial reduction in feed intake when an animal is first offered a bulky diet is a reflection that the GIT has not yet adapted to the new diet. Once adaptation has been reached and the GIT is able to accommodate increased gut fill or the increased involvement of the parts of the GIT involved in fiber digestion, new feed intake equilibrium can be reached. It is therefore important to improve our understanding of the rate of adaptation to bulky diets and how this is affected by factors such as age and prior experience to bulky ingredients.

Birds sometimes consume less when they are offered an unfamiliar food, a behavior termed as neophobia (Marples and Kelly, 1999; Bertin et al., 2010), which may confer evolutionary advantages, such as avoidance of a potentially harmful substance. The birds in this experiment were switched over to the bulky diets after feeding on the starter diets for 7 d, which did not contain any of the subsequent bulky ingredients. The switch to the bulky diets was abrupt, so it is possible that the feed intake on the bulky diets could be a response to their novelty. However, the expectation would be that a neophobic response to novel foods should not be associated with their composition per se; any such response should not be associated with the amount of a bulky ingredient in the diet, which was the case here where diets contained different amounts of the same bulky ingredient. Most of the birds (on 7 out of the 8 treatments) were also switched over from their bulky diets to a novel, bulkier diet (GM60) during Period 2 of the experiment. Their intake on this diet increased almost instantaneously (in some cases within a day) and reflected the degree of the prior adaptation of the birds on the bulky diets. Therefore, the concept of neophobia cannot account for the initial response of the birds on the ‘novel’ bulky diets of this experiment.

We conclude that at least in the short term, the dietary WHC does seem to be a good predictor of SFI when birds are initially introduced to a bulky diet, consistent with Nascimento et al. (2020). However, it seems that once the birds have adapted to the bulkiness of the diet, we can no longer use dietary WHC to predict feed intake since the diet is no longer limiting. Furthermore, since the plasticity of the GIT is seemingly greater in younger birds, the age at which broilers are introduced to a bulky diet may also have implications on the accurate prediction of feed intake, although this warrants further investigation. Identifying links between hydration capacities, such as dietary WHC, with both physical and chemical characteristics of diets could be used to further improve the accuracy of predictions of voluntary feed intake and performance. It should be noted that the levels of inclusion used in this experiment are far greater than what would be used under practical situations, as they were chosen to develop predictive relationships under a wide range of circumstances. The results suggest that there would be some considerable flexibility in the use of alternative ingredients that may affect the feed intake of birds through their nutrient density.

## Appendix Supplementary materials

Image, application 1

## Footnotes

Supplementary material associated with this article can be found in the online version at doi:10.1016/j.psj.2021.101501.
