# Supplementary material for: Towards the prediction of feed intake capacity of modern broilers on bulky feeds
Source: Poult Sci. 2021 Sep 24;100(12):101501. doi: 10.1016/j.psj.2021.101501 (PMC8554256; doi:10.1016/j.psj.2021.101501)
Supplement: Supplementary file 1 [file mmc1.docx]

| **Chemical composition (%) ^a^** | OH | WB | GM |
| --- | --- | --- | --- |
|  |  |  |  |
| Metabolizable energy (kcal kg^-1^) (calculated) | 1075 | 1600 | 1123 |
| Crude protein (CP) | 3.10 | 14.95 | 9.60 |
| Lysine (calculated) | 0.03 | 0.63 | 0.37 |
| Crude fat (oil A)^b^ | 1.33 | 3.90 | 1.90 |
| Total oil (oil B)^c^ | 1.67 | 4.43 | 2.65 |
| Ash | 3.30 | 5.60 | 3.80 |
| Calcium (calculated) | 0.19 | 0.14 | 0.59 |
| Available Phosphorus (calculated) | 0.18 | 0.27 | 0.30 |
| Crude fibre | 20.3 | 12.8 | 22.0 |
| Neutral detergent fibre | 36.4 | 38.6 | 44.0 |
| Acid detergent fibre | 32.0 | 13.0 | 28.6 |
| Acid detergent lignin | 4.95 | 3.18 | 6.13 |
| Density (g/ ml) | 1.73 | 1.68 | 2.09 |
| Water Holding Capacity (g/ g DM) | 3.60 | 4.60 | 7.49 |
| ^a^ Analysed composition unless otherwise stated | | | |
| ^b^ Ether extractable portion of fat ^c^ Total fat in the sample | | |  |

Table S1. Chemical composition (%), density (g ingredient/ ml water displaced) and water holding capacity (g of water/ g of ingredient DM) of the bulky ingredients used to dilute the diets offered to broiler chickens (oat hulls, OH; wheat bran, WB; and grass meal, GM).
